# Multiple Cortical Hypointense Lesions Revealed by T2*-Weighted Imaging and Micro-aneurysmal Lesions in a Case of Cardiac Myxoma

**DOI:** 10.7759/cureus.107061

**Published:** 2026-04-14

**Authors:** Atsushi Shima, Daisuke Kambe, Jyuji Takeuchi, Yutaka Kobayashi

**Affiliations:** 1 Department of Neurology, Kyoto Kizugawa Hospital, Kyoto, JPN; 2 Department of Neurology, Kyoto University Graduate School of Medicine, Kyoto, JPN; 3 Department of Neurosurgery, Kyoto Kizugawa Hospital, Kyoto, JPN; 4 Department of Cardiovascular Surgery, Uji Tokushukai Medical Center, Kyoto, JPN

**Keywords:** microbleeds, mitral valve, stroke, t2*, tumor

## Abstract

Multiple cortical hypointense lesions on T2*-weighted images and ischemic lesions are one of the radiological manifestations of cardiac myxoma, while the mechanism of the hypointense lesions is inconclusive. We describe a 60-year-old woman with a cardiac myxoma who presented with multiple ischemic lesions and cortical hypointense lesions revealed by T2*-weighted images. A gadolinium-enhanced T1-weighted image revealed micro-aneurysmal lesions, which corresponded to the hypointense lesions. Previous pathological studies have revealed that aneurysmal lesions could be caused by cardiac myxoma. Our findings suggest a potential association between cortical microbleeds and microaneurysmal changes in patients with cardiac myxoma.

## Introduction

Cardiac myxoma is the most common primary cardiac tumor in adults, though it remains a relatively rare cause of ischemic stroke overall [1]. Embolic events, including ischemic strokes, occur in a significant proportion of patients with left-sided myxomas. In addition to ischemic events, hemorrhagic manifestations, such as intracerebral hemorrhages, microbleeds, and micro-aneurysms, are also well-documented neurological complications [2-4]. However, the exact pathophysiological mechanisms underlying these hemorrhagic lesions - particularly multiple scattered cortical hypointense lesions seen on T2*-weighted imaging (T2*WI) - remain inconclusive [5]. It is hypothesized that embolic tumor cells invade the arterial wall, leading to microaneurysm formation and subsequent microhemorrhages, yet direct in vivo radiological evidence remains scarce. Here, we report a case of cardiac myxoma presenting with transient neurological symptoms, multiple acute ischemic lesions, and scattered cortical hypointense lesions on 1.5-Tesla T2*WI. Furthermore, we highlight the clinical novelty of this case by illustrating the radiological relationship between these hypointense lesions and micro-aneurysmal lesions detected by contrast-enhanced magnetic resonance imaging (MRI).

## Case presentation

**Figure 1:**
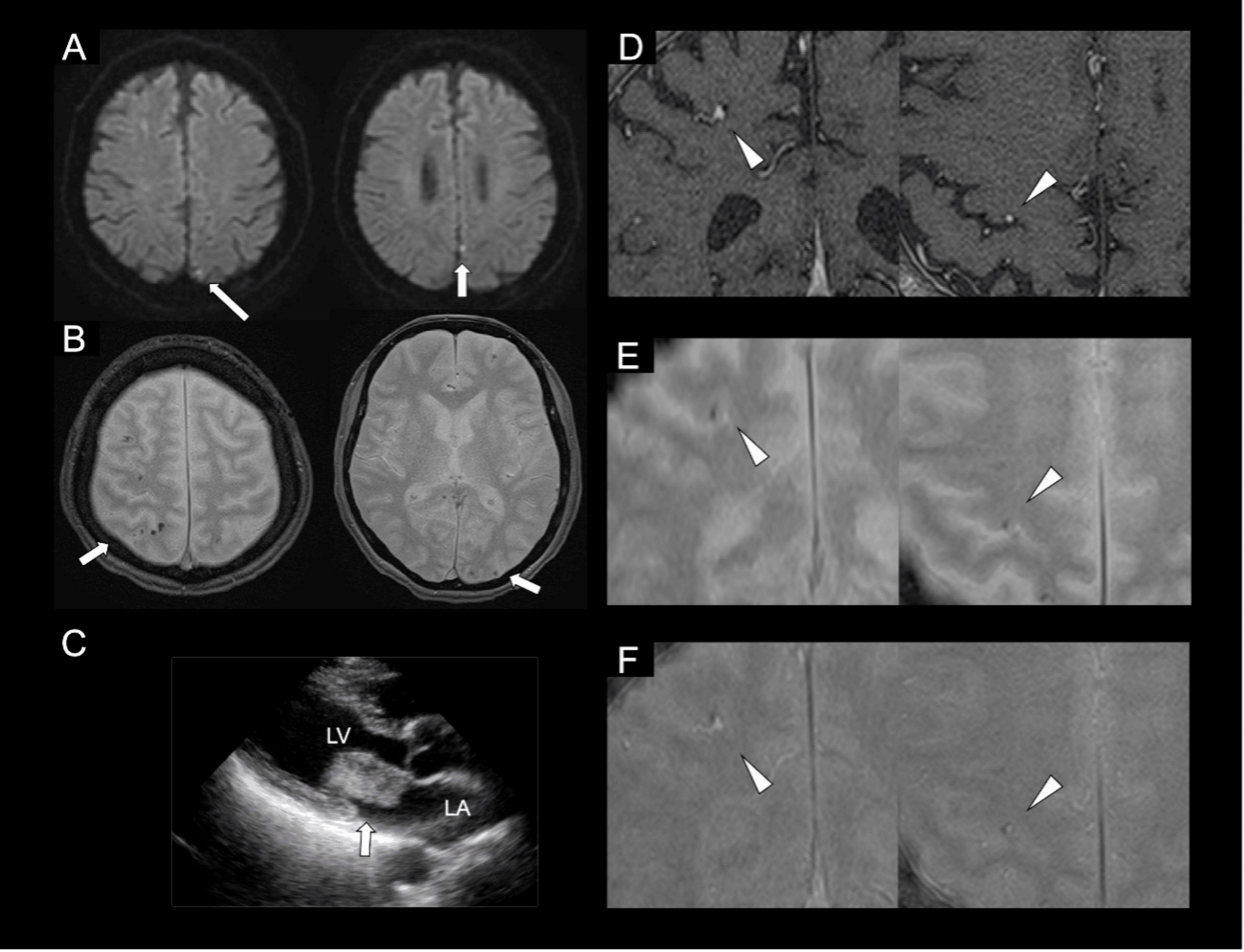
Brain Magnetic Resonance Imaging and Echocardiography Findings in a Case of Cardiac Myxoma (A) Diffusion-weighted images elucidate multiple acute ischemic lesions (arrows). (B) T2*WI reveals multiple scattered cortical hypointense lesions (arrows). (C) Transthoracic echocardiography reveals an oscillating tumor (arrow) on the mitral valve. (D) Gadolinium-enhanced T1-weighted image shows micro-aneurysmal lesions (arrowheads). (E) T2*WI reveals the hypointense lesions (arrowheads) corresponding to the micro-aneurysmal lesions. (F) Fusion image of gadolinium-enhanced T1-weighted image and T2*WI corresponding to micro-aneurysmal lesions (arrowheads). LV: left ventricle; LA: left atrium.

A 60-year-old woman presented to the emergency department with a sudden onset of transient left upper limb weakness. Upon initial evaluation, the symptoms had already fully resolved, and the neurological examination revealed no focal neurological deficits. On the same day, a 1.5-Tesla magnetic resonance imaging (MRI) scan of the brain was performed. Diffusion-weighted images revealed multiple ischemic lesions, and T2*-weighted imaging (T2*WI) detected multiple scattered cortical hypointense lesions. A gadolinium-enhanced T1-weighted image showed several micro-aneurysmal lesions corresponding to the hypointense lesions revealed by T2*WI (Figures 1A, 1B, 1D, 1E). No stenotic lesions of the cerebral or carotid arteries were revealed by magnetic resonance angiography. Atrial fibrillation was not detected by Holter electrocardiography.

Subsequent outpatient evaluation was planned to further investigate the cause of the multiple infarctions. Five days after the initial presentation, an oscillating tumor on the mitral valve, measuring 3 cm in diameter, was identified via transthoracic echocardiography (Figure 1C), which was highly suspicious for a cardiac myxoma.

The patient was then referred to the cardiovascular surgery department at another hospital. Fourteen days after the initial presentation, the patient underwent surgical resection of the tumor, and the postoperative pathological examination confirmed the diagnosis of a benign cardiac myxoma. The surgical procedure was completed successfully without any perioperative complications. Following the surgery, the patient's neurological status remained completely stable, with no appearance of new neurological symptoms suggestive of recurrent ischemic stroke or hemorrhage. She was discharged home in good condition. After discharge, she was followed up at the cardiovascular surgery outpatient clinic for several visits. She remained asymptomatic without any recurrence of cardiovascular or cerebrovascular events, and her outpatient follow-up was subsequently concluded.

## Discussion

Multiple cortical hypointense lesions revealed by T2*WI are the characteristic findings for cerebral amyloid angiopathy, infective endocarditis, and moyamoya disease. A pivotal study previously demonstrated that T2*WI hypointense spots can identify microcerebral aneurysms in patients with infective endocarditis [6]. Given the similar embolic pathophysiology, scattered cerebral hypointense lesions are also reported as manifestations of cardiac myxoma [5]. Vanacker et al. described hypointense lesions detected by T2*WI as cortical microbleeds that were negative for [11C] PiB positron emission tomography in the case of an ischemic stroke caused by cardiac myxoma, which led to the conclusion that the scattered microbleeds were caused by myxoma and not cerebral amyloid angiopathy [2]. One possible mechanism for the detection of hypointense lesions by T2*WI was reported to be a consequence of minor hemorrhage related to microaneurysms, as suggested by cerebral angiography findings [1,3]. These embolic tumor cells are thought to penetrate the endothelium, destroy the internal elastic lamina, and proliferate within the subintimal space, leading to the structural weakening of the vessel wall and subsequent aneurysmal dilation. In the present case, the hypointense lesions observed by T2*WI partially corresponded to the micro-aneurysmal lesions detected by contrast-enhanced MRI (Figures 1D-1F). The aneurysmal lesions related to myxoma were pathologically confirmed by an autopsied case report to be caused by a tumor invasion of the arterial wall [1,4]. However, rather than definitively proving causation, our findings suggest a potential association between cortical microbleeds and microaneurysmal changes in patients with cardiac myxoma. Recognizing these specific radiological signatures using 1.5-Tesla T2*WI and contrast-enhanced MRI is imperative. It aids not only in the differential diagnosis of multiple cortical microbleeds but also in the early detection of hidden vascular complications, allowing for safer long-term management.

## Conclusions

In conclusion, we encountered a case of cardiac myxoma presenting with multiple ischemic lesions alongside multiple cortical hypointense spots on T2*WI. The precise anatomical correlation between these hypointense spots and micro-aneurysms on contrast-enhanced MRI suggests a potential association between cortical microbleeds and microaneurysmal changes. Careful evaluation utilizing T2*WI is crucial for identifying these vascular complications in patients with cardiac myxoma.
